# Arteritic Anterior Ischemic Optic Neuropathy in Takayasu Arteritis: An Ominous Systemic Sign?

**DOI:** 10.18502/jovr.v20.14442

**Published:** 2025-07-30

**Authors:** Kaviyapriya Natarajan, Amit Kumar Deb, Hemanth Ramachandar, Disha Agarwal, Augustine Jose, Shreyas Temkar

**Affiliations:** ^1^Department of Ophthalmology, Jawaharlal Institute of Postgraduate Medical Education and Research (JIPMER), Puducherry, India; ^2^Department of Clinical Immunology, Jawaharlal Institute of Postgraduate Medical Education and Research (JIPMER), Puducherry, India

**Keywords:** Immunosuppression, Ischemic Optic Neuropathy, Retinopathy, Takayasu Arteritis, Vasculitis

## Abstract

**Purpose:**

To report a case of a patient diagnosed with arteritic anterior ischemic optic neuropathy, who was later diagnosed with a severe form of Takayasu arteritis (TA).

**Case Report:**

A 34-year-old lady presented with a sudden, painful loss of vision in the left eye for four months associated with headache and jaw claudication. Ocular examination revealed features suggestive of arteritic anterior ischemic optic neuropathy and microaneurysms scattered throughout the fundus in both eyes, consistent with Takayasu retinopathy. General examination and investigations, including CT aortogram, confirmed the diagnosis of TA. Despite being put on maximal immunosuppression, she developed severe systemic manifestations within the next three months and passed away due to the illness.

**Conclusion:**

AION as a presenting feature in the setting of TA is uncommon, and its occurrence may indicate a progressive course and poor systemic outcomes.

##  INTRODUCTION

Takayasu arteritis (TA) is a rare, chronic, granulomatous large-vessel vasculitis that typically appears in the second to third decades of life and is significantly more prevalent in females than in males. The aorta and its primary branches are affected by the inflammatory process. Different phenotypical clusters have been proposed based on distinct patterns of arterial involvement.^[1]^ The disease course in TA often fluctuates, characterized by alternating periods of exacerbation and remission, which requires long-term immunosuppression. Only about 20% of patients are reported to have a monophasic, self-limiting disease course.^[2]^ Ocular symptoms occur in nearly 45% of patients and typically manifest as the disease progresses.^[3,4]^ TA's most common ocular manifestation is Takayasu retinopathy, while anterior ischemic optic neuropathy (AION) without additional ocular findings is uncommon.^[4]^ Here, we report the case of a 34-year-old female who initially presented with features of arteritic AION, was later diagnosed with TA, and ultimately passed away due to severe systemic manifestations of the illness.

##  CASE PRESENTATION 

A 34-year-old female presented with a complaint of sudden, painful loss of vision in the left eye for the past four days. This complaint was associated with a headache, predominantly over the left hemicranium, with the maximum intensity located over the left temple, along with jaw claudication. She reported several episodes of syncope and transient visual obscuration over the past month. The patient also provided a history of upper limb claudication, significant weight loss, and loss of appetite for the last six months. There was no previous history of oral ulcers, hair loss, rashes, fever, limb weakness, or bowel and bladder issues. Her past medical history was significant for a recent diagnosis of type 2 diabetes mellitus, which was managed with oral hypoglycemic agents. Additionally, she reported menorrhagia, for which she was prescribed cyclical oral contraceptive pills.

**Figure 1:**
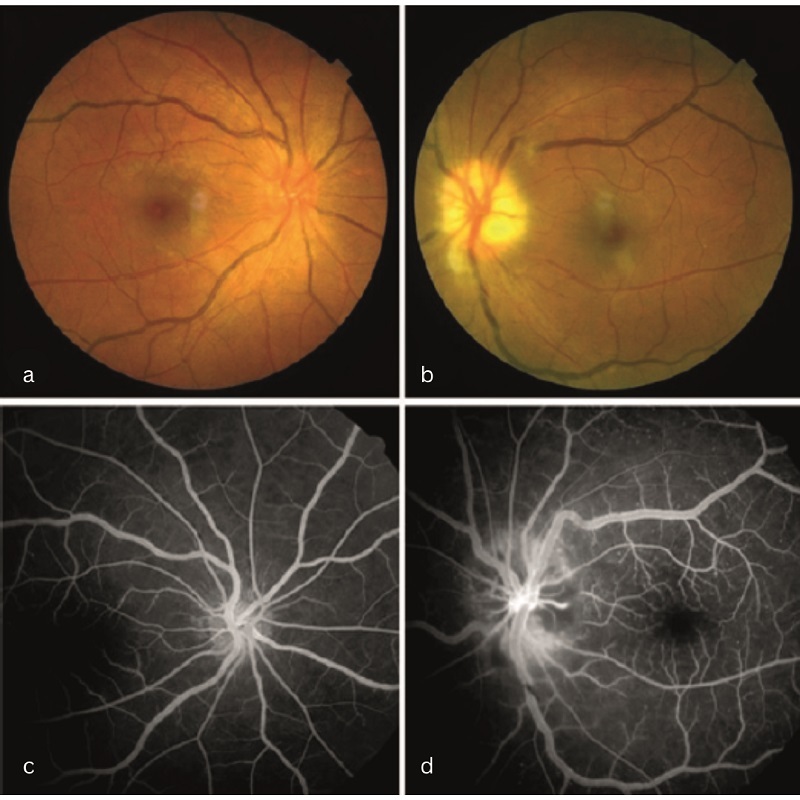
Fundus examination showing a small, crowded, hyperemic disc in the right eye (a), and pallid disc edema in the left eye (b), with dilated and tortuous vessels in both eyes. (c & d) FFA showing segmental filling defects of the disc and the adjacent choroid, suggestive of ischemic optic neuropathy in the left eye and microaneurysms in both eyes.

On ocular examination, visual acuity was 6/6 in the right eye and finger counting (CF) close to the face with accurate light projection in all quadrants of the left eye. The left eye showed a grade three relative afferent pupillary defect. The anterior segment evaluation was within normal limits. Fundus examination revealed a small, crowded, hyperemic disc in the right eye, alongside pallid disc edema in the left eye, with dilated and tortuous vessels present in both eyes [Figures 1a & 1b]. Fundus fluorescein angiography (FFA) showed segmental filling defects in the disc and the adjacent choroid, suggestive of ischemic optic neuropathy in the left eye and microaneurysms scattered throughout the fundus in both eyes [Figures 1c & 1d]. Visual evoked potentials (VEPs) showed significantly decreased N2-P2 amplitudes with normal latency in the left eye. Based on the clinical appearance and FFA, we established a diagnosis of Takayasu retinopathy with arteritic AION. To support this clinical diagnosis, we conducted a thorough general examination, which revealed the presence of pallor (anemia) and a right carotid bruit. The pulse was weak, and blood pressure was unrecordable in the upper limbs. A blood pressure reading of 170/100 mmHg was noted at the level of the popliteal artery in the lower limbs. The remainder of the systemic examination was normal, further supporting the diagnosis of TA. Laboratory investigations showed neutrophilic leukocytosis (12,500 cells/mm^3^), thrombocytosis (500,000 platelets/mm^3^), elevated ESR (96 mm/hour), and a high C-reactive protein level (18 mg/dL).

**Figure 2:**
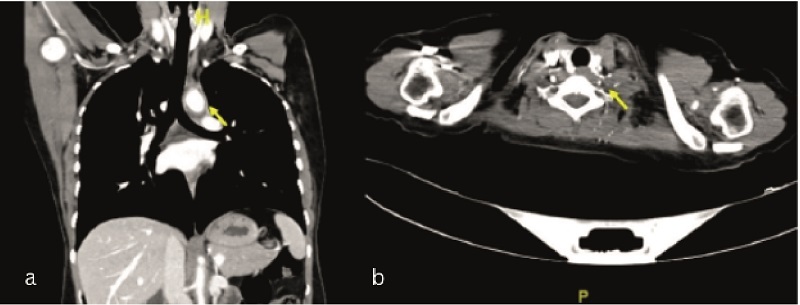
CT aortogram showing thickening and vascular occlusion of the thoracic aorta (a) and left vertebral artery (b) in coronal and axial scans, respectively, as marked by the arrows.

A transthoracic echocardiogram recorded normal left ventricular function and indicated no aortic regurgitation or root dilation. Magnetic resonance imaging and an angiogram of the brain revealed multiple chronic lacunar infarcts along with severe narrowing of the left internal carotid and left vertebral arteries. A CT aortogram showed diffuse, circumferential, long-segment thickening and vascular occlusion involving the thoracic aorta, the left internal carotid artery, the left vertebral artery, and the bilateral subclavian arteries [Figures 2a & 2b].

The patient was classified as a case of TA based on the criteria proposed by the American College of Rheumatology in 1990.^[5]^ Her disease severity score, as recorded by the Indian Takayasu Arteritis Score-A, was 28. Given the severe manifestations, she was administered intravenous methyl prednisolone at a dose of 1 gram per day for three days, followed by oral prednisolone at a dose of 1 mg/kg daily along with dual antiplatelet drugs. Monthly intravenous cyclophosphamide therapy was subsequently initiated.

**Figure 3:**
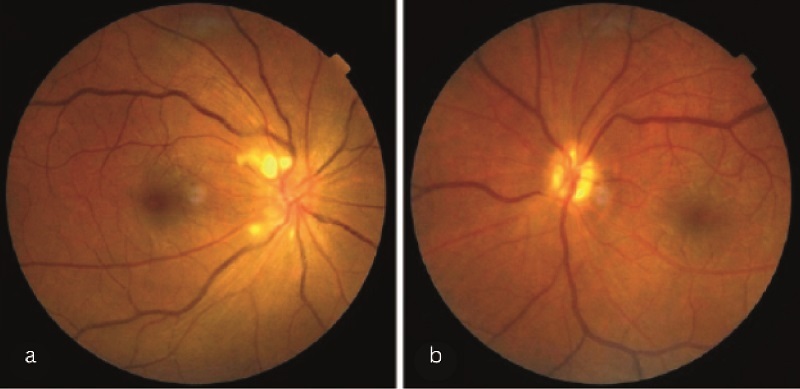
Fundus examination showing peripapillary cotton wool spots in the right eye (a) and secondary optic atrophy in the left eye (b), three months after the initial presentation.

Three months after the initial presentation, the patient returned with diminution of vision in the right eye for a week, accompanied by focal seizures and new weakness affecting the left upper limb and right lower limb. Examination revealed a visual acuity of 6/12 in the right eye and CF in the left eye. Additionally, fundus examination demonstrated peripapillary cotton wool spots in the right eye and secondary optic atrophy in the left eye [Figures 3a & 3b]. Furthermore, neuroimaging indicated new infarcts in the brain and extensive filling defects in the cranial sinuses, suggestive of cerebral venous thrombosis. Anti-phospholipid antibodies were not detected, and the thrombotic manifestation was presumed to have been caused by the use of oral contraceptive pills. Consequently, she was scheduled to be switched to tocilizumab therapy; however, her condition subsequently worsened, resulting in status epilepticus and uncontrolled hypertension, which led to her demise. Alongside the deterioration of the patient's systemic condition, bilateral severe ocular hypotony was observed secondary to severe carotid occlusion.

##  DISCUSSION

TA is a rare inflammatory large-vessel vasculitis that predominantly affects the aorta and its branches, as well as the pulmonary and coronary arteries. Common systemic findings include reduced or absent pulses, hypertension, vascular bruits, congestive cardiac failure, and transient ischemic attacks.^[3]^

Among ocular findings, retinal involvement is most commonly reported in TA, occurring in 13.5% to 35% of patients.^[3]^ It is noteworthy that approximately 75% of TA diagnoses are made after patients develop ocular issues.^[6]^ Ocular manifestations in TA can either be disease-related or treatment-related. Disease-related ocular features typically arise from carotid artery involvement, leading to ocular hypoperfusion and manifesting as Takayasu retinopathy or ischemic optic neuropathy. Alternatively, ocular changes may occur as a secondary effect of systemic hypertension due to renal artery stenosis, presenting as hypertensive retinopathy.^[5]^

In a review by Szydełko-Paśko et al, the most common symptoms included a gradual reduction in visual acuity (52.5%), sudden visual loss (23%), ocular pain (17.2%), and amaurosis fugax (25.4%). The most prevalent retinal manifestations were retinal ischemia (57.4%), followed by ischemic optic neuropathy (18%), cataract (14.8%), and retinal artery occlusion (12.3%).^[6]^ Notably, although ocular ischemia is common in TA, the occurrence of AION, especially as a presenting feature, is relatively rare.^[4]^

Although the disease course is often unpredictable, TA is associated with significant morbidity and mortality. A multicentric study by Mirouse et al on 318 patients with TA reported an overall mortality rate of 5% at the end of a 6.1-year follow-up.^[7]^ Common causes of TA mortality include heart failure, renal failure, pulmonary infections, postoperative complications, and cerebrovascular accidents.^[8]^ Several prognostic factors have been identified in the literature. These factors include a lower baseline body mass index (BMI), younger age at presentation, a progressive clinical course, thoracic aorta involvement, stroke, and elevated CRP; all of which are associated with poor outcomes and necessitate more aggressive management.^[9,10]^ In their assessment of prognostic indicators in TA, Comarmond et al found a higher incidence of vascular complications (83%) and increased 5-year mortality in patients with retinopathy.^[10]^ Our patient presented with arteritic AION as the initial presenting feature and retinopathy, later identified as having systemic vascular involvement. Despite prompt and aggressive treatment, her systemic condition deteriorated over a period of four months. Notable poor prognostic factors in this case included low baseline BMI, a progressive disease course, and retinopathy. In our opinion, the occurrence of arteritic AION alongside Takayasu retinopathy may serve as a potential indicator of poor systemic health prognosis.

In summary, TA can present with a wide range of presenting features, often posing a diagnostic challenge for ophthalmologists and physicians. Although arteritic AION is an uncommon presenting feature of TA, its occurrence may indicate a more aggressive disease course and be associated with poor systemic outcomes.

##  Financial Support and Sponsorship

None.

##  Conflicts of Interest

None.
